# (Cost-)Effectiveness of personalised multimodal physiotherapy compared to surgery in patients with painful cervical radiculopathy. Protocol for a randomised non-inferiority trial (The MOVE-IT study)

**DOI:** 10.1016/j.conctc.2026.101626

**Published:** 2026-03-09

**Authors:** Florine E. Marinelli, Ivo J. Lutke Schipholt, Sebastiaan Klein Heerenbrink, Michel W. Coppieters, Johanna M. van Dongen, Raymond W.J.G. Ostelo, Sjoerd C. Kielstra, Carmen L.A. Vleggeert-Lankamp, Mark P. Arts, Bastiaan Ter Meulen, Hans J.L.W. Bosboom, Femke van Nassau, Pieter Coenen, Servan Rooker, Marije L.S. Sleijser-Koehorst, MOVE-IT Research Group, Carine den Boer, Gert Joan Bouma, Linda Bralten, Yvette Grimbergen, Diederik Kempen, Maarten Liedorp, Germine Mochel, Willem Oerlemans, Martijn Pruissen, Krista Roon, Niki Schoonenboom, Esther Verstraete, Leen Voogt, Tom van de Voort, Gwendolyne G.M. Scholten-Peeters

**Affiliations:** a Department of Human Movement Sciences, Faculty of Behavioural and Movement Sciences, Vrije Universiteit Amsterdam, Amsterdam Movement Sciences - Program Musculoskeletal Health, van der Boechorststraat 9, 1081 BT, Amsterdam, the Netherlands; b Fysiotherapie Utrecht Oost, Bloemstraat 65D, 3581 WD, Utrecht, the Netherlands; c School of Allied Health, Sport and Social Work, Griffith University, 4111, Brisbane (Nathan), Australia; d Department of Health Sciences, Amsterdam Movement Sciences, Faculty of Science, Vrije Universiteit Amsterdam, De Boelelaan 1085, 1081 HV, Amsterdam, the Netherlands; e Department of Epidemiology and Data Science, Amsterdam Movement Sciences, Amsterdam UMC, Vrije Universiteit Amsterdam, De Boelelaan 1117, 1081 HV, Amsterdam, the Netherlands; f Department of Neurosurgery, Leiden University Medical Center, Albinusdreef 2, 2333 ZA, Leiden, the Netherlands; g Department of neurosurgery, Haaglanden Medisch Centrum, Lijnbaan 32, 2512 VA, The Hague, the Netherlands; h Department of Neurology, OLVG, Oosterpark 9, 1091 AC, Amsterdam, the Netherlands; i Department of Public and Occupational Health, Amsterdam Public Health Research Institute, Amsterdam UMC, De Boelelaan 1117, 1081 HV, Amsterdam, the Netherlands; j Department of Neurosurgery and Orthopaedics, ViaSana clinic, Hoogveldseweg 1, Mill, 5451 AA, the Netherlands; k Department of Family Medicine and Population Health (FAMPOP), University of Antwerp, Wilrijkstraat 10, 2650, Edegem, Belgium

**Keywords:** Cervical radiculopathy, Neuropathic pain, Neck pain, Rehabilitation, Physiotherapy, Surgery, Randomised controlled trial

## Abstract

**Background:**

Painful cervical radiculopathy can lead to substantial and long-lasting limitations in activities and participation. Surgery is considered when conservative treatment fails to deliver relevant improvements or when neurological signs are severe. However, personalised multimodal physiotherapy may offer non-inferior outcomes to surgery, with possibly fewer adverse events and lower costs. Further research is needed to assess the (cost-)effectiveness of personalised multimodal physiotherapy compared to surgery.

**Methods:**

This randomised non-inferiority study compares personalised multimodal physiotherapy to anterior cervical discectomy with fusion (1:1 allocation ratio) among 126 patients with painful cervical radiculopathy having an indication for surgery, recruited by neurologists. Personalised multimodal physiotherapy uses a mechanism-based approach within a biopsychosocial framework and is tailored to the individual patient. The primary outcome is disability over 12 months using the neck disability index with a prespecified non-inferiority margin of three points. Secondary outcomes include arm and neck pain, fear of movement, and complications. Outcomes are measured at baseline and at three, six, nine, and 12 month follow-up. Additionally, a process evaluation and cost-effectiveness analysis will be performed. Data will be analysed according to the ‘intention-to-treat’ and the 'per-protocol' principle. Both will be conducted using linear and logistic mixed models.

**Ethics and dissemination:**

The study is approved by the medical ethics committee Brabant (P2327).

**Study registration number and status:**

The study protocol is registered at Open Science Framework (ID:S7HWA; registered June 27th, 2023). Recruitment commenced in May 2024. All data are anticipated to be collected by July 2027 when data analysis and interpretation will commence.

## Highlights

• First trial evaluating (cost-)effectiveness of personalised multimodal physiotherapy versus surgery in painful CR.
• The personalised physiotherapy treatment is developed using latest insights in treatment of painful CR.
• This approach is unique and may offer a (cost-)effective and safe treatment alternative for people with painful CR.
• Conducting a process evaluation will provide insights into potential selection bias from patient treatment preferences.
• Evaluating the development and delivery of the personalised multimodal physiotherapy treatment may enhance its implementation

## 1 Background

Painful cervical radiculopathy (CR) is a form of specific neck pain, in which a cervical nerve root and/or dorsal root ganglion is mechanically and/or chemically irritated. It is caused by narrowing of the foraminal space due to spondylarthrosis and/or disc herniation [[1], [2], [3]]. Radiculopathy refers to cervical nerve root-related neurological deficits, such as sensory, motor or reflex changes, while radicular pain describes the pain that results from nerve root compression or irritation [4]. Radiculopathy and radicular pain can occur in isolation, but often coexist, which is then referred to as painful CR [4]. Painful CR negatively impacts physical function, social participation and mental health [1,3]. In the Netherlands, around 2800 surgeries for painful CR are performed yearly with direct costs estimated at €30 million annually [5,6]. With even substantially higher indirect costs (e.g. due to productivity loss), painful CR imposes a significant societal burden [5].

The natural and clinical course of painful CR is highly variable [2,7]. Symptoms resolve largely over 4-6 months, and ∼80% of patients recover within 24-36 months [8,9]. However, if conservative management does not lead to meaningful improvements, patients are usually referred to a neurologist and invasive treatments are considered, such as epidural steroid injections and/or surgery [10,11]. Anterior Cervical Discectomy with Fusion (ACDF) is the most common surgical approach for people with painful CR and is generally considered effective at reducing arm pain [12]. Although surgery provides faster pain relief than conservative management, the long-term effect of personalised multimodal physiotherapy may be comparable [[13], [14], [15]] and total costs may be lower. Also, the potential adverse events associated with ACDF surgery are avoided with conservative management.

Personalised multimodal physiotherapy integrates interventions proven to reverse pathophysiological mechanisms linked to entrapment neuropathies [[16], [17], [18]]. The pathophysiology of painful CR remains not fully understood [19], but involves a complex interplay of peripheral and central mechanisms. The interactions between these mechanisms and the impact of psychosocial factors underscore the importance of addressing both peripheral and central mechanisms, as well as psychosocial factors, in the treatment of painful CR. Physiotherapy that targets these mechanisms has shown effectiveness in reducing pain and disability [[16], [17], [18]], especially when tailored to individual needs [20]. A recent systematic review revealed that improvements in pain and disability following personalised multimodal physiotherapy were comparable to surgery for people with painful CR at 12 months follow-up [14]. However, this review only included two relatively small studies, and the certainty of the evidence was low. Furthermore, no cost-effectiveness studies have been conducted. Therefore, based on the assumed comparable effectiveness [14], fewer and less severe adverse-events and lower costs associated with personalised multimodal physiotherapy, we will conduct a non-inferiority study to assess the (cost-)effectiveness and process of implementation of personalised multimodal physiotherapy compared with ACDF surgery for people with painful CR. We hypothesise that personalised multimodal physiotherapy is non-inferior to ACDF surgery, with fewer and less severe adverse-events, and is associated with lower healthcare and societal costs. This paper describes the study design and outlines the content of personalised multimodal physiotherapy.

## 2 Methods and design

### 2.1 Study design

This is a pragmatic multicentre non-inferiority randomised clinical trial (RCT) with parallel economic and process evaluation. To describe the treatment The Standard Protocol Items: Recommendations for Interventional Trials (SPIRIT) statement [21] will be followed and the Template for Intervention Description and Replication (TIDier) checklist is used [22].

### 2.2 Study population

Patients with painful CR with an indication for surgery and no contra-indication for physiotherapy are eligible to participate. The diagnosis of painful CR requires two criteria: (1) a clinical presentation indicative of painful CR and (2) relevant corresponding findings on MRI (e.g., disc herniation and/or spondylosis). The clinical presentation consists of radicular pain (i.e., pain due to irritation of a cervical nerve root or dorsal root ganglion [14]) with typically more intense arm pain than neck pain [23], and at least one of the following: paraesthesia, numbness, muscle weakness, and/or reduced reflexes [1,2,24]. Additional inclusion criteria are age ≥18, and at least eight weeks of unilateral arm pain with an intensity of ≥4 on a 0-10 Numeric Rating Scale (NRS). Exclusion criteria are cervical myelopathy, motor deficits measured with the Medical Research Council (MRC) scale for muscle strength ≤3, previous neck surgery, contra-indication for surgery, inability to perform physical activity [25], current self-reported psychiatric disorders, rheumatic disease (rheumatoid arthritis, spondylitis ankylopoetica, Forestier's disease), malignancies causing CR-like complaints, or pregnancy.

### 2.3 Procedure

#### 2.3.1 Screening phase

**Fig. 1:**
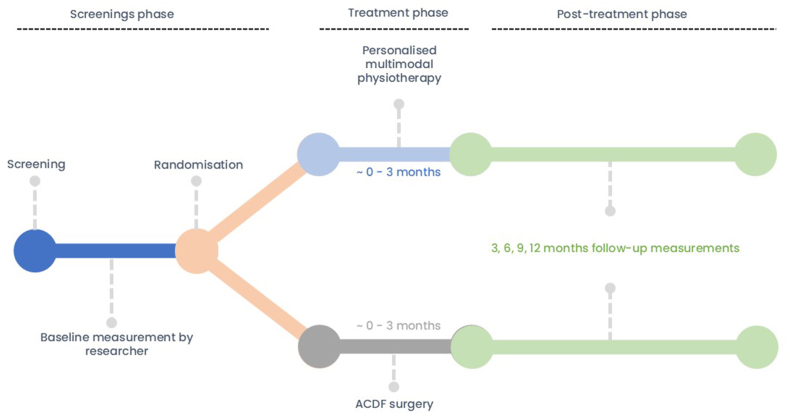
Flowchart of the different study phases. Abbreviations: ACDF: Anterior cervical discectomy with fusion.

Potential participants are informed about the study by neurologists from 11 secondary care hospitals and clinics, who diagnoses painful CR. A neurosurgeon verifies whether there is an ACDF surgery indication. Patients who are eligible and provide written informed consent will be scheduled for baseline measurements. Baseline questionnaires are filled in by the participants, and physical tests are conducted by a clinical investigator (FM, SKH or SK). In Fig. 1 the study flow and different study phases are shown.

#### 2.3.2 Randomisation and blinding

After baseline measurement, an in independent researcher randomly allocates participants via a phone call to personalised multimodal physiotherapy or surgery with a 1:1 allocation ratio using concealed, computer-generated block randomisation (block sizes of four or six) [26]. Due to the nature of the study, blinding of patients and health care providers is not feasible. However, the clinical investigators and the statistician conducting the analyses are blinded.

#### 2.3.3 Treatment phase

In the treatment phase, the physiotherapy group receives personalised multimodal physiotherapy. In case of progressive neurological deficits, or a substantial increase in arm pain or paraesthesia, participants are referred back to the neurologist for further assessment, at which point a (delayed) ACDF surgery could be decided. The surgery group receives the ACDF surgery conform usual care.

#### 2.3.4 Follow-up time-points

Follow-up assessments will take place at three, six, and 12 months. To reduce the risk of recall bias in cost reporting, an additional cost evaluation will be conducted at nine months. Participants who have not completed the follow-up questionnaire will receive reminders on days five and eight. If responses are still missing after two weeks, a follow-up phone call will be made to encourage adherence to the study.

### 2.4 Treatment

#### 2.4.1 Personalised multimodal physiotherapy

Personalised multimodal physiotherapy is a tailored mechanism-based treatment approach that combines multiple interventions based on the individual needs, symptoms, and goals of each patient within a biopsychosocial model [20]. The overall aims of this treatment are to restore function, enhance the patient's ability to manage their condition independently, and reduce pain.

Based on current knowledge of working mechanisms, the interventions used within personalised multimodal physiotherapy are divided into two categories: treatments with a predominantly peripheral or central mechanistic effect, while acknowledging that various interventions (e.g., exercise) influence both pain mechanisms [[27], [28], [29]]. Peripheral pain mechanisms include changes in ectopic nerve impulses (e.g. reducing local neuroinflammation [30,31]) and changes in nociceptor activity (e.g. muscle spasm). Central pain mechanisms occur within the spinal cord or brain and include central processing of nociceptive signals and the modulation of descending inhibitory and facilitatory activity. Selecting interventions that specifically address the underlying physiology of a particular pain mechanism may enhance the effectiveness of physiotherapy [18]. Table A1 (Appendix) provides an overview of pain mechanisms and suggested interventions.

**Fig. A1:**
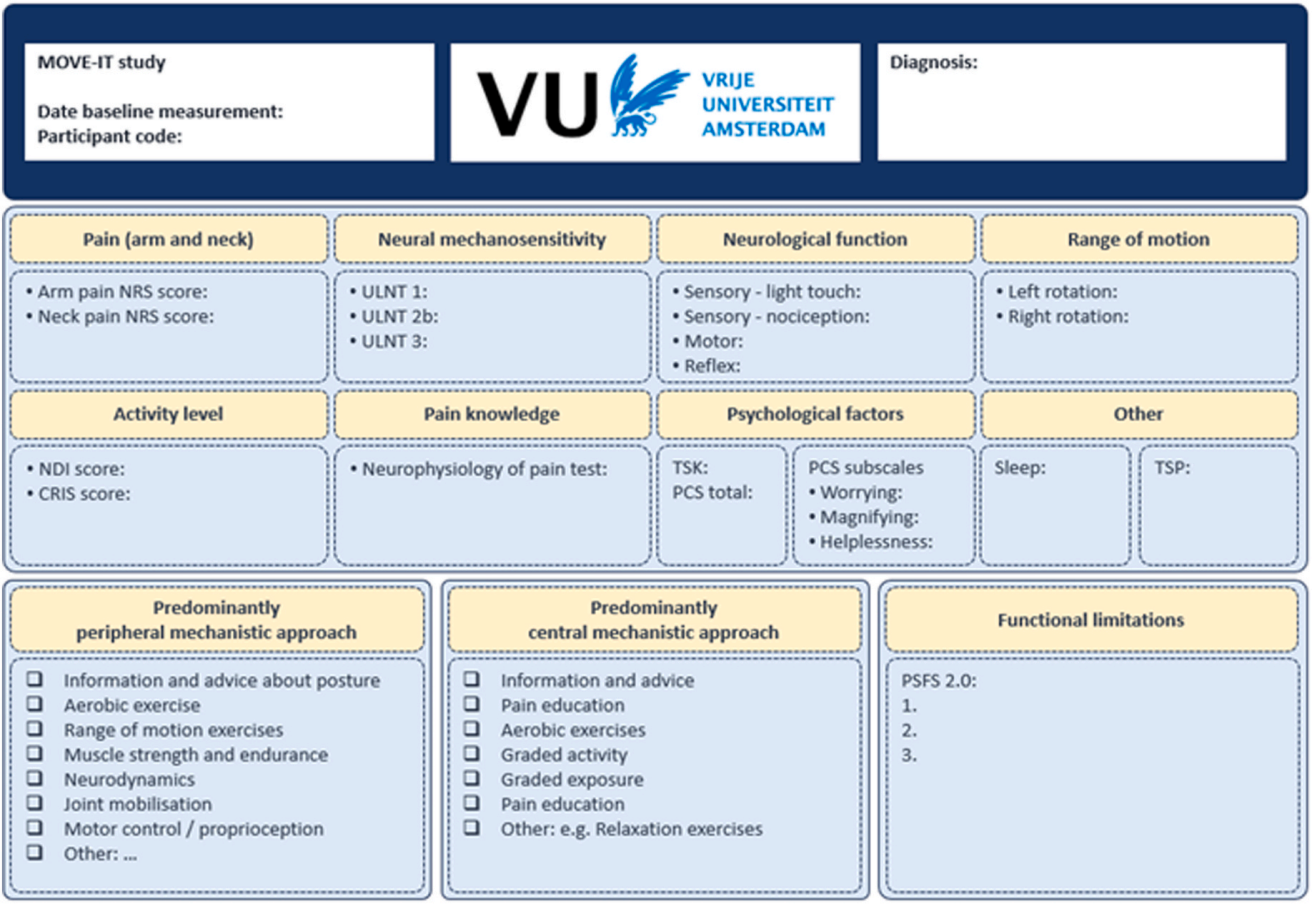
Assessment of baseline characteristics and treatment recommendations. Abbreviations: NDI: Neck Disability Index, NRS: Numeric Rating Scale, CRIS: Cervical Radicular Impact Scale, TSK: Tampa Scale of Kinesiophobia, PCS: Pain Catastrophising Scale, PSFS 2.0: Patient-Specific Functioning Scale 2.0, ULNT: Upper Limb Neurodynamic Test, CROM: Cervical Range of Motion, TSP: Temporal Summation of Pain.

Over a period of eight to 12 weeks, the physiotherapy will take place without a set number of treatment sessions. Personalised multimodal physiotherapy is delivered in live, one-on-one sessions within the existing care structure at the physiotherapists' clinics. The baseline measurement results are compiled into a visual diagram (see Figure A1), which is shared with the treating physiotherapists and forms the basis for selecting the intervention. During the intake session, the physiotherapist and patient collaboratively establish treatment goals through shared decision-making. Adjustments to the treatment plan are made in subsequent sessions when necessary.

Some participants may experience mild side effects following physiotherapy, such as neck discomfort, muscle stiffness, fatigue, increased muscle tension, or flare-up of symptoms. These rarely last longer than 24 h [32]. There are no reported severe side effects of the proposed physiotherapy treatment [7,[16], [17], [18],^33^].

##### 2.4.1.1 Participating physiotherapists

Physiotherapists who hold a master's degree in musculoskeletal physiotherapy are eligible to deliver the physiotherapy treatment. During a one-day training, working mechanisms of interventions are explained, techniques are practiced, case-based training is provided to enhance practical decision-making skills and a manual is distributed describing relevant theory. Due to the personalised nature of the treatment, physiotherapists use their clinical expertise to determine the appropriate frequency and duration, rather than following a fixed schedule and personalise interventions within protocol guidelines. A guideline for therapy intensity is the resolution of treatment-related pain and/or symptom exacerbation within 24-36 h. Physiotherapists are instructed to monitor each individual treatment session and document the details of the interventions provided. Quality assurance is maintained through regular communication with physiotherapists and evaluation of their adherence to the protocol. Additionally, physiotherapists can seek expert guidance from two professionals of the research team (GSP, ILS) who have over ten years of relevant experience.

#### 2.4.2 ACDF surgery

ACDF surgery is performed by experienced neurosurgeons in participating hospitals, conform usual care. In summary, after general anaesthesia the surgeon makes a small transverse skin incision at the left or right side of the neck, according to the preference of the surgeon [34,35]. Medial to the carotid sheath, the pre-vertebral space is opened and the anterior cervical spine is exposed. The correct index level is verified with fluoroscopy. Caspar spreader and two distraction pins are placed in the affected segment. Care will be taken not to damage the adjacent level discs. A standard anterior discectomy with the aid of loupe magnification or microscope (depending on the surgeon's preference) is performed. The posterior longitudinal ligament is opened and the nerve root and dura are decompressed adequately. Once the anterior discectomy has been performed, the endplates are prepared for fusion. According to the preference of the surgeon, a solid titanium cage or polyetheretherketone interbody cage (filled with local bone obtained from removed osteophytes) is placed within the intervertebral disc space under fluoroscopic guidance. Depending on the surgeon's preference and patient characteristics an anterior fixation plate can be placed to secure the cage. If required, a vacuum drain is placed and the wound is closed in layers.

Most hospitals have a follow-up six to nine weeks after surgery [10,35] and recommend performing minimal home exercises, taking pain medication (e.g., paracetamol [35]), and following lifestyle guidelines. Post-operative physiotherapy is not advised during recovery [10].

A systematic review reported adverse events for ACDF ranging from 13.2 to 19.3% in the United States [36]. The most frequent complication was transient dysphagia [36]. However, studies conducted in the Netherlands indicate that complication rates are lower, ranging from 0 to 16% [37,38].

#### 2.4.3 Cross-over

The participant and physiotherapist will evaluate the effectiveness of the treatment, based on persistence of symptoms. If deemed unsuccessful or in case of progressive increase of symptoms, a delayed ACDF surgery remains an option and can be initiated at any time during the follow-up period of the study. Also, if complaints are in remission, ACDF surgery can be cancelled. Due to the pragmatic study design, co-interventions are allowed in both treatment arms.

### 2.5 Outcome measures

Patients complete online surveys using Qualtrics to collect data (Qualtrics, Amsterdam, The Netherlands; www.qualtrics.com). All physical tests are performed in-person by the clinical investigators. An overview of timing of data collection and measuring tools is provided in Table 1.

**Table 1:** Overview and timing of measurements and measuring tools.

Domain	Questionnaire	T0	T1	T2	T3	T4
Disability in people with neck pain	Neck Disability Index (NDI)	x	x	x	-	x
Pain intensity	Numeric Rating Scale (NRS) for arm and neck pain	x	x	x	-	x
Perceived recovery	Global Perceived Effect (GPE)	-	x	x	-	x
Quality of life	EuroQol 5 Dimension 5 Level (EQ-5D-5L)	x	x	x	-	x
Symptoms and limitations	Cervical Radicular Impact Scale (CRIS)	x	x	x	-	x
Activity limitations	Patient Specific Functional Scale (PSFS 2.0)	x	x	x	-	x
Patient acceptable symptom state	Patient Acceptable Symptom State (PASS)	x	x	x	-	x
Fear of Movement	Tampa Scale of Kinesiophobia (TSK)	x	x	x	-	x
Pain catastrophizing	Pain Catastrophizing Scale (PCS)	x	x	x	-	x
Knowledge of pain neurophysiology	Neurophysiology of pain test	x	x	-	-	-
Economic evaluation	Cost questionnaire	x	x	x	x	x
Sociodemographic and clinical data	Custom-made questionnaire	x	x	x	x	x

**Domain**	**Physical test**					

Cervical range of motion	Left and right rotation	x	-	-	-	x
Muscle strength	Kenn muscles C5-Th1	x	-	-	-	x
	C5: m. deltoideus					
	C6: m. biceps brachii					
	C7: m. triceps brachii caput longum					
	C8: m. adductor pollicis					
	Th1: mm. interossei palmaris and dorsalis					
	Th2: n.a.					
Nerve mechano-sensitivity	ULNT 1 (median), 2B (radial) and 3 (ulnar)					
Sensibility (light touch)	Soft brush C5-Th2	x	-	-	-	x
Sensibility (nociception)	Pinwheel C5-Th2					
Reflex	Biceps and triceps tendon reflex	x	-	-	-	x
Pain modulation (facilitation)	Temporal Summation of Pain (C6 and TA)	x	-	-	-	x

Abbreviations: ULNT: Upper Limb Neurodynamic Test, TA: tibialis anterior. Details about the scores and clinimetric properties are presented in Table A2 (Appendix). T0: baseline, T1: 3 months, T2: 6 months, T3: 9 months, T4: 12 months.

#### 2.5.1 Primary outcomes

The primary outcome is disability over 12 months, measured with the Neck Disability Index (NDI) [39], which assesses self-reported pain intensity and limitations of activities in daily life. The Dutch version of the NDI is a valid and responsive measure of disability [40].

#### 2.5.2 Secondary outcomes

The secondary outcomes are arm and neck pain intensity (NRS), perceived recovery (GPE) [40], quality of life (EQ-5D-5L) [41], symptoms and limitations (CRIS) [42], patient-specific activity limitations (PSFS 2.0) [43], patient acceptable symptom state (PASS) [44], fear of movement (TSK) [45], pain catastrophising (PCS) [46], knowledge of pain mechanisms (neurophysiology of pain test) [47], return-to-work (days), medication use, healthcare and societal costs (cost questionnaire), percentage of cross-overs, treatment adherence, (re)surgeries and complications.

### 2.6 Sample size and non-inferiority margin

A sample size of 126 participants is needed to achieve a power of 0.80 to test the hypothesis that personalised multimodal physiotherapy is non-inferior to ACDF surgery, with a standard deviation of six points on the NDI, an alpha of 2.5%, and allowing for a drop-out rate of up to 20% [48,49].

To determine the non-inferiority margin, we calculated the Smallest Worthwhile Effect (SWE) for the Neck Disability Index (NDI). The SWE represents the minimum clinical benefit required to justify a treatment, accounting for its specific risks, costs, and burdens [50,51]. While a 20% between-group difference is typically recommended for conservative treatments [50], this threshold is often increased by 10% for chronic conditions [52].

In consultation with our patient representative, we set the SWE at 30%. Given that the Minimal Clinically Important Change (MCIC) for patients undergoing surgery for cervical radiculopathy is nine points on the NDI [53], the 30% threshold translates to 3 points non-inferiority margin. Consequently, physiotherapy is considered non-inferior to surgery if the difference in NDI improvement between the two groups is less than three points.

### 2.7 Economical evaluation

The economic evaluation will be performed from a societal and healthcare perspective, according to the Dutch Guideline for Economic Evaluations [54]. Societal costs will include all costs related to the interventions under study irrespective of who pays or benefits, such as the cost of the prescribed treatment, other healthcare use, informal care, and paid and unpaid productivity losses (including both absenteeism and presenteeism). Healthcare costs only include costs accruing to the standard Dutch healthcare sector. Treatment costs will be micro-costed. All other costs will be measured via cost questionnaires, and valued using the Dutch manual for costing studies in healthcare [55]. The EQ-5D-5L health states will be converted into utility scores using the Dutch tariff for the EQ-5D-5L [56]. Quality-adjusted Life Years (QALYs) will be estimated via the ‘Area under the Curve’ approach.

### 2.8 Process evaluation

A process evaluation of the delivery of the personalised multimodal physiotherapy will be conducted in accordance with the UK MRC guidelines on both patient and therapist level [57]. Several research objectives have been formulated using the mechanisms of treatment and are identified in a logic model. These include contextual barriers and facilitating factors for implementing the treatment (i.e., reach of the target population and delivery of the treatment by investigating dose delivered/received and fidelity) and mechanisms of impact (i.e., did the treatment work as intended). We will contact participants and physiotherapists by email to inquire whether they would like to participate in the focus groups and/or (semi-structured) interviews. To collect data, several questions regarding treatment satisfaction at three months follow-up, (semi-structured) interviews and focus groups will be conducted with physiotherapists and patients from both interventions. Also, field notes and routinely collected data will be used for the process evaluation. All data will be collected during or shortly after the study. A written standard operating procedure will be used to ensure consistency and quality during data collection.

### 2.9 Analyses

#### 2.9.1 Statistical analysis

First, normality of the data will be checked by the Kolmogorov-Smirnov test and visual inspection of Q-Q plots, box plots, and histograms. If needed, data will be transformed to obtain normal residuals. The primary analysis will be conducted according to the intention-to-treat principle, with secondary analyses performed based on the per-protocol approach. A one-sided alpha of 2.5% will be used. For the primary and secondary outcomes, linear mixed models will assess between-group differences in outcome measures over 12 months. To analyse the over-time effect, the fixed part of the model will include the outcome measure over time, treatment group, and baseline outcome correction (when applicable). A random intercept will be selected to account for the correlated nature of multiple measurements from the same participant. Potential confounders will be included as covariates in the adjusted model. To assess the differences in treatment effect at each time point, we will include an interaction term between time and treatment group. The regression coefficient (B), p-value, and confidence intervals (95% CI) will be computed for the crude models (only corrected for the baseline value of the outcome) and the confounder-adjusted models.

#### 2.9.2 Non-inferiority evaluation

Differences between the study-arms with 95% confidence interval and p-values will be calculated for the crude and adjusted models based on potential differences in patient characteristics with a non-inferiority margin of three points on the NDI over 12 months. Missing data and reasons for drop-outs will be reported. Linear/logistic mixed models handle missing data more flexibly due to its reliance on maximum likelihood estimation, which allows for the incorporation of missing values under missing at random assumptions.

#### 2.9.3 Cost-effectiveness analyses

The economic evaluations will be performed for the primary effect measure ‘disability’ and QALYs. The Incremental Cost-Effectiveness Ratio (ICER) will be calculated by dividing the difference in costs across study arms by the corresponding difference in effects. These differences will be estimated using regression techniques that will account for one or more of the following statistical challenges: baseline imbalances, skewed costs and/or effects, correlated costs and effects, clustering of data and missing data, if applicable [58]. Incremental cost-effect pairs will be plotted on cost-effectiveness planes, and cost-effectiveness acceptability curves will be constructed. The latter indicate the probability of personalised multimodal physiotherapy being cost-effective in comparison to surgery for various values of willingness to pay (i.e., the maximum amount that decision-makers are willing to pay per unit of effect gained). Sensitivity analyses will be performed to assess the robustness of the results (e.g., complete-case analysis and per protocol).

#### 2.9.4 Process evaluation

Descriptive statistics will be used to analyse the quantitative part, and a thematic content analysis for the qualitative part (i.e., mixed-methods design).

### 2.10 Patient and public involvement

A panel of four people with painful CR co-developed the study design, research questions, choice of treatment groups, and patient burden. Two of these people and two representatives from the public reviewed the patient information letter, and their feedback was used to further improve the letter.

### 2.11 Data, integrity, and management

A data management plan is set up to ensure findable, accessible, interoperable, and reusable (FAIR) use of data [59]. Collected data consist of signed informed consent, digitally provided information, and physical test reports (either online or on paper). All data will be de-identified using a unique participant number. The data obtained from the samples are exported directly to a research drive only accessible to employees of the research team at the Vrije Universiteit Amsterdam. To ensure data quality, data constraints are pre-programmed in the online questionnaire. Further, frequent checks of coherent reporting are conducted. Clinical investigators assessing outcomes are trained to conduct the test in a standardised way and use a fixed protocol.

### 2.12 Ethics and dissemination

The study will be conducted according to the principles of the Declaration of Helsinki (2013) and the Medical Research Involving Human Subjects Act (WMO). The research team will process the data, and both positive and negative findings will be disclosed unreservedly. Results will be submitted for publication to peer-reviewed scientific journals. Individual de-identified participant data that underlie the results will be shared. Investigators whose proposed data use has been approved may access the data for individual participant data meta-analysis.

The study is approved by the Medical Ethics Committee (METC) Brabant which can be found under the reference number P2327 and is preregistered at the Open Science Framework (OSF) under S7HWA (https://doi.org/10.17605/OSF.IO/S7HWA) at June 27th, 2023. In case of important protocol changes an amendment will be submitted for approval to the METC Brabant.

## 3 Discussion

This paper describes the protocol for a non-inferiority randomised clinical trial that will assess the (cost-)effectiveness of personalised multimodal physiotherapy compared to ACDF surgery over a 12-month period in patients with painful CR who have an indication for surgery. We hypothesise that personalised multimodal physiotherapy is non-inferior to ACDF surgery in patients with painful CR based on disability over one year.

There is a strong need for studies investigating the effectiveness of personalised multimodal physiotherapy [14]. To our knowledge, this is the first study that simultaneously investigates the effectiveness and cost-effectiveness of personalised multimodal physiotherapy compared to surgery in patients with painful CR who have an indication for surgery. The personalised multimodal physiotherapy treatment is unique and carefully developed based on prior research into the effectiveness of the interventions for painful CR [[60], [61], [62]]. We believe the design of this study allows to assess whether the proposed treatment is a feasible alternative to ACDF surgery, which is the golden standard for painful CR and generally regarded as effective in reducing pain associated with this condition [12,13,[63], [64], [65]]. The non-inferiority design allows for flexibility in the treatment choice for people with painful CR, by demonstrating that personalised multimodal physiotherapy might be comparable effective on disability whilst offering additional benefits that might align better with individual preferences.

Besides the strengths, the proposed study has some potential limitations. The use of a non-inferiority margin is inherently prone to discussion. When comparing conservative treatment to no-treatment, the between-group difference should be at least 20% [50]. In this study, however, we compare conservative treatment to surgical treatment. As surgery is an invasive treatment associated with higher risks for (serious) adverse events, and based on the smallest worthwhile effect for chronic sciatica complaints [52], we increased the threshold to 30%. This results in a non-inferiority margin of 3 points on the NDI. Further, the use of the NDI as primary outcome measure doesn't take into account the radicular arm pain, which is one of the most important symptoms people with painful CR experience [12,23].

The results of this study will contribute to a better understanding of (cost-)effectiveness of personalised multimodal physiotherapy for painful cervical radiculopathy, and might offer a good alternative treatment option to surgery. Depending on the findings, multifaceted implementation strategies will be developed (e.g., educational) to facilitate implementation at different levels (e.g., healthcare professionals and guidelines).

## CRediT authorship contribution statement

**Florine E. Marinelli:** Writing – review & editing, Writing – original draft. **Ivo J. Lutke Schipholt:** Writing – review & editing, Methodology, Funding acquisition, Conceptualization. **Sebastiaan Klein Heerenbrink:** Writing – review & editing. **Michel W. Coppieters:** Writing – review & editing, Methodology, Funding acquisition, Conceptualization. **Johanna M. van Dongen:** Writing – review & editing, Methodology, Conceptualization. **Raymond W.J.G. Ostelo:** Writing – review & editing, Methodology, Conceptualization. **Sjoerd C. Kielstra:** Writing – review & editing. **Carmen L.A. Vleggeert-Lankamp:** Writing – review & editing, Methodology, Conceptualization. **Mark P. Arts:** Writing – review & editing. **Bastiaan Ter Meulen:** Writing – review & editing. **Hans J.L.W. Bosboom:** Writing – review & editing. **Femke van Nassau:** Writing – review & editing, Methodology, Conceptualization. **Pieter Coenen:** Writing – review & editing, Methodology. **Servan Rooker:** Writing – review & editing. **Marije L.S. Sleijser-Koehorst:** Writing – review & editing, Methodology, Funding acquisition, Conceptualization. **Gwendolyne G.M. Scholten-Peeters:** Writing – review & editing, Methodology, Formal analysis, Conceptualization.

## Funding statement

The study is funded by The Dutch Association for Manual Therapy (NVMT) grant number NVMT-91122. The NVMT did not play a role in the design of the study, and will not play a role in the analysis or reporting of the findings.

## Declaration of competing interest

The authors declare that they have no known competing financial interests or personal relationships that could have appeared to influence the work reported in this paper.

## Figures and Tables

**Table A1:** Factors and mechanisms with therapy options

*Factors and mechanism*	Therapy options
Central mechanisms	

*Pain (arm and neck)*	• (Aerobic) exercise • Pain education • Neurodynamic treatment • Manual therapy

*Pain knowledge*	• Pain education

*Psychological factor:* *Worrying, magnifying, helplessness*	• Aerobic exercises • Graded activity • Graded exposure • Pain education

*Psychological factor:* *Fear/pain avoidance*	• Aerobic exercises • Graded activity • Graded exposure • Pain education

*Sleep*	• Aerobic exercises • Sleep and pain education (aspects of cognitive behavioural approach)

***Peripheral mechanisms***	

*Mechano-sensitivity*	• Neurodynamic treatment • Aerobic exercise

*Mobility*	• Neurodynamic treatment • Motor control exercises • Manual therapy • Aspect from a cognitive behavioural approach

*Other: Muscle strength/endurance*	• Motor control exercises • Muscle strength • Neurodynamic treatment • Manual therapy

**Table A2:** Questionnaires and their clinical properties

Questionnaire	Aim	Info	Clinimetric properties
Modified Neck Disability Index (NDI): arm pain and neck pain	Assess self-reported pain intensity and disabilities in daily work and activities	**NoQ:** 16 (4 generic, 6 specific for respectively arm and neck pain)	Valid and responsive measure of disability for standard version [39,40]
		**Score:** 0-50. Multiply final score to get percentage	
		**Int.:** Higher score indicates more pain and greater disability	
Numeric Rating Scale (NRS) arm pain	Assess mean pain intensity last 24 h	**NoQ:** 1	Valid and reliable tool to assess pain intensity in people painful CR [66]
		**Score:** 0 (no pain) to 10 (worst possible pain)	
		**Int.:** Higher score indicates more pain	
Numeric Rating Scale (NRS) neck pain	Assess mean pain intensity last 24 h	**NoQ:** 1	Valid and reliable tool to assess pain intensity in people painful CR [66]
		**Score:** 0 (no pain) to 10 (worst possible pain)	
		**Int.:** Higher score indicates more pain	
Global Perceived Effect (GPE)	Assess perceived recovery	**NoQ:** 1	Valid and reliable tool for assessing health transitions in people with musculoskeletal disorders [67,68] Very good-to-excellent test-retest reliability in people with neck pain [69]
		**Score:** a 7-point Likert Scale	
		**Int.:**	
EQ-5D-5L	Assess quality of life across 5 domains	**NoQ:** 6 (5 descriptive, 1 VAS)	Valid, reliable and feasible instrument to assess preference-based health status in a wide range of conditions [56,70]
		**Score:** a 5-point Likert scale and a VAS	
		**Int.:** the final score is a 5-digit code representing a health state and is converted to an index value specific for the country/region to estimate quality of life	
Cervical Radicular Impact Scale (CRIS)	Assess improvement in symptoms and functionality of the arm and neck in people with painful CR	**NoQ:** 21 items divided over 3 subscales with resp. 9 (symptoms) 6 (energy and posture) and 6 (actions and activities) items	Good content validity, test-retest reliability, construct validity and is able to discriminate between groups [42]
		**Score:** a 5-point Likert scale	
		**Int.:** sum scores can be calculated and converted to predict EQ-5D utility (see Ref. [42])	
Modified Patient Specific Functional Scale 2.0 (PSFS 2.0)	Assess activity limitations	**NoQ:** 3-5, with optional 2 extra	Considered to be valid in terms of content validity and construct validity for patients with neck pain and [65]
		**Score:** 0 (impossible to perform activity) to 10 (fully possible to perform activity)	
		**Int.:** higher score means better functioning	
Patient Acceptable Symptom State (PASS)	Assess the clinical relevance of a treatment effect	**NoQ:** 1	Reliable and consistent for a given condition in a non-changing setting [44,71]
		**Score:** binary scale (yes or no)	
		**Int.:**	
Tampa Scale of Kinesiophobia (TSK)	Assess fear of movement	**NoQ:** 17	Valid and reliable questionnaire in Dutch [45] that is the preferred self-administrated questionnaire to assess fear of movement in musculoskeletal pain [46]
		**Score:** 4 point scale ranging from ‘strongly disagree’ to ‘strongly agree’	
		**Int.:** Total score can be between 17 and 68. A score of ≤37 is interpreted as absence of fear of movement, a score of >37 is interpreted as fear of movement	
Neurophysiology of pain test	Assess the understanding and knowledge of patients on the neurophysiology of pain	**NoQ:** 13	Considered to be valid [72]
		**Score:** true, false, unknown	
		**Int.:** To investigate if the score changed due to intervention	
Pain Catastrophising Scale	Assess catastrophic thinking associated with pain	**NoQ:** 13 items	Preferred self-administrated questionnaire to assess pain catastrophising in musculoskeletal pain [46]
		**Score:** 5 point scale with end points ‘not at all’ to ‘all the time’, scoring between 0 and 52	
		**Int.:** A score higher than 24 indicates catastrophic thinking	

Abbreviations: NoQ: Number of questions; Int.: Interpretation of score. VAS: Visual Analogue Scale.

## Data Availability

No data was used for the research described in the article.
